# Reply to Dietvorst et al. Challenges in Providing an Overview of Results of Intermittent Fasting Interventions on Diabetes Parameters. Comment on “Silva et al. Effects of Intermittent Fasting on Regulation of Metabolic Homeostasis: A Systematic Review and Meta-Analysis in Health and Metabolic-Related Disorders. *J. Clin. Med.* 2023, *12*, 3699”

**DOI:** 10.3390/jcm13144094

**Published:** 2024-07-12

**Authors:** Ana Inês Silva, Manuel Direito, Filipa Pinto-Ribeiro, Paula Ludovico, Belém Sampaio-Marques

**Affiliations:** 1 Life and Health Sciences Research Institute (ICVS), School of Medicine, University of Minho, 4710-057 Braga, Portugal; a92562@alunos.uminho.pt (A.I.S.); a92640@alunos.uminho.pt (M.D.); filiparibeiro@med.uminho.pt (F.P.-R.); pludovico@med.uminho.pt (P.L.); 2 ICVS/3B’s-PT Government Associate Laboratory, 4806-909 Guimarães, Portugal

We deeply value and appreciate the insightful feedback provided by the authors of the comment [1] on our systematic review titled “Effects of Intermittent Fasting on Regulation of Metabolic Homeostasis: A Systematic Review and Meta-Analysis in Health and Metabolic-Related Disorders”, authored by Silva et al. in 2023 [2]. Before commenting on the arguments raised by authors considering our review incomplete, we have to stress that it seems awkward that in Table 1, the authors have listed at least six papers that were published in 2023 and eighteen in 2022 (probably in the second half of 2022, although it is not possible to be sure given that references were not given), when we have stated, according to the guidelines, that our search was performed between November 2021 and June 2022. Even if we met the criteria the authors demand we would have not detected these papers. This seems an unfair assessment that should never have been published without clarification. This is not the same period of time as we have considered in our review.

## In Response to the Arguments Raised in the Comment to Our Paper

### The Different forms and Definitions of Intermittent Fasting

As outlined in the search strategy for our paper, we opted to exclude articles involving any other dietary interventions besides intermittent fasting. We defined intermittent fasting as the voluntary abstinence from food and drink for a specific period of time [3]. While we acknowledge that this exclusion criterion resulted in the omission of a significant number of papers, we believe it was necessary to ensure the relevance and integrity of our systematic review for its intended purpose. Fasting mimicking diets (FMDs) are a group of miscellaneous protocols that, while sharing some properties with intermittent fasting, are not as defined as true fasting protocols. We considered these to be a potential confounding factor for the analysis. This was not the focus of our systematic review, as clearly stated in our manuscript, and, therefore, the inclusion of FMDs was beyond the scope of our study.

### A Lack of Alternative Search Strategies

We employed Mesh Terms along with multiple databases to ensure a thorough search process, following the guidelines. We were confident that this approach, combined with the application of our exclusion criteria, would enable us to identify the majority of relevant original papers. Moreover, it is worth highlighting that our manuscript underwent peer review, during which the adequacy of the search strategies employed was carefully evaluated and validated.

### Selection of Outcomes

The outcomes selected for our meta-analysis aimed to provide a comprehensive overview of the various metabolic states of individuals. Metabolic disease impacts the cell’s ability to perform critical biochemical reactions, affecting processes such as the transport of proteins, carbohydrates, or lipids [4]. Additionally, these conditions increase the risk of diabetes *mellitus* type 2 (T2D), obesity and hypertension. Our main aim was to compare how different metabolic conditions, such as obesity, T2D and metabolic syndrome (MetS), are influenced by intermittent fasting compared to healthy individuals. To achieve this, we evaluated key outcome measures, including weight, BMI, HDL, total cholesterol, triglycerides, glucose and insulin, among other, that are relevant to all the conditions studied [5,6,7]. While we recognize the importance of more specific parameters, such as those mentioned in the comment, it is important to note that these may not be consistently reported across studies assessing conditions other than diabetes. Furthermore, with regard to HbA1c, it is crucial to acknowledge that this analysis represents an average glucose level over the past three months. Given the variability in study durations, this parameter may not provide a reliable basis for comparison across different studies [8,9].

### The Exclusion Criteria

A balance of specific inclusion and exclusion criteria is paramount in any systematic review or even in clinical trials. The relevance of inclusion and exclusion criteria in the design of studies ensures that researchers have clear guidelines for selecting subjects and determining which submissions will receive attention for review. In any study, the establishment of exclusion criteria will leave out studies.

In summary, we would like to reiterate that the primary objective of our review was to understand the effects of intermittent fasting on various metabolic conditions, rather than focusing specifically on diabetes. Therefore, our review is not incomplete, although it does not meet the criteria defined by other authors. We consider that the raised arguments are subjective, and it seems that the authors would have carried out a different systematic review with different criteria.
